# Supplementary material for: Design and analysis of a plasmonic-nanorod-enhanced lead-free inorganic perovskite/silicon heterojunction tandem solar cell exceeding the Shockley–Queisser limit
Source: RSC Adv. 2025 Oct 31;15(50):42254–71. doi: 10.1039/d5ra05323d (PMC12577752; doi:10.1039/d5ra05323d)
Supplement: RA-015-D5RA05323D-s001 [file RA-015-D5RA05323D-s001.pdf]

Supplementary Information (SI) for RSC Advances.
This journal is © The Royal Society of Chemistry 2025

# Supplementary Information

# Design and Analysis of Plasmonic-Nanorod-Enhanced Lead-Free Inorganic Perovskite/Silicon Heterojunction Tandem Solar Cell Exceeding the Shockley-Queisser Limit

Md. Sad Abdullah Sami[1], Arpan Sur[1] and Ehsanur Rahman[1]*

[1] Department of Electrical and Electronic Engineering, Bangladesh University of Engineering and Technology, Dhaka, 1000, Bangladesh

*Email: ehsaneee@eee.buet.ac.bd

## S1. Material Data for Device Simulation

The material data utilized in the electrical simulation of both the top and bottom cells is presented in tables S1 and S2, respectively.

**Table S1:** Electrical parameters of top cell materials used in simulation

| Features | FTO (n) | $TiO_2$ (n) | $CsSnGeI_3$ (p) | $Cu_2O$ (p) |
|---|---|---|---|---|
| Permittivity | 9 | 10 | 9 | 7.1 |
| Bandgap (eV) | 3.2 | 3.2 | 1.5 | 2.17 |
| Electron affinity (eV) | 4.4 | 4 | 3.9 | 3.2 |
| Conduction band effective density of states ($cm^{-3}$) | $2.2\times10^{18}$ | $1\times10^{21}$ | $2.2\times10^{18}$ | $2\times10^{17}$ |
| Valence band effective density of states ($cm^{-3}$) | $1.8\times10^{19}$ | $2\times10^{20}$ | $1.8\times10^{19}$ | $1.1\times10^{19}$ |
| Electron thermal velocity (cm/s) | $1\times10^{7}$ | $1\times10^{7}$ | $1\times10^{7}$ | $1\times10^{7}$ |
| Hole thermal velocity (cm/s) | $1\times10^{7}$ | $1\times10^{7}$ | $1\times10^{7}$ | $1\times10^{7}$ |
| Electron mobility ($cm^2V^{-1}s^{-1}$) | 90 | 20 | 974 | 200 |
| Hole mobility ($cm^2V^{-1}s^{-1}$) | 90 | 10 | 213 | 80 |
| Acceptor concentration ($cm^{-3}$) | - | - | $10^{14}$ | $10^{17}$ |
| Donor concentration ($cm^{-3}$) | $10^{19}$ | $10^{18}$ | - | - |
| SRH (lifetime in seconds) | $10^{-8}$ ($e^-$)<br>$10^{-8}$ ($h^+$) | $10^{-7}$ ($e^-$)<br>$10^{-8}$ ($h^+$) | $10^{-7}$ ($e^-$)<br>$10^{-7}$ ($h^+$) | $10^{-8}$ ($e^-$)<br>$10^{-7}$ ($h^+$) |
| Radiative recombination (electron-hole pairs capture rate $cm^3/s$) | - | - | $10^{-10}$ | $10^{-10}$ |
| References | [1], [2] | [2], [3], [4] | [1], [2], [3] | [1], [2], [4] |

**Table S2:** Electrical parameters of bottom cell materials used in simulation

| Features | a-Si (n/p) | GaSb (n) | c-Si (n) |
|---|---|---|---|
| Permittivity | 11.7 | 15.7 | 11.7 |
| Bandgap (eV) | 1.7 | 0.72 | 1.11452 |
| Electron affinity (eV) | 3.9 | 4.06 | 4.05 |
| Conduction band effective density of states ($cm^{-3}$) | $1\times10^{20}$ | $2.1\times10^{17}$ | $2.8\times10^{19}$ |
| Valence band effective density of states ($cm^{-3}$) | $1\times10^{20}$ | $1.8\times10^{19}$ | $1.04\times10^{19}$ |
| Electron mobility ($cm^2V^{-1}s^{-1}$) | 10 | 3000 | 1471 |
| Hole mobility ($cm^2V^{-1}s^{-1}$) | 1 | 1000 | 470.5 |
| Acceptor concentration ($cm^{-3}$) | $10^{19}$ | - | 0 |
| Donor concentration ($cm^{-3}$) | $10^{17}$ | $10^{16}$ | $10^{16}$ |
| SRH (lifetime in seconds) | $10^{-12}$ ($e^-$) $10^{-13}$ ($h^+$) | $10^{-07}$ ($e^-$) $10^{-07}$ ($h^+$) | $10^{-06}$ ($e^-$) $10^{-06}$ ($h^+$) |
| Radiative recombination (electron-hole pairs capture rate $cm^3/s$) | - | $10^{-10}$ | $10^{-14}$ |
| Auger recombination (carrier capture coefficient $cm^6/s$) | - | - | $2.8\times10^{-31}$ ($e^-$) $1\times10^{-31}$ ($h^+$) |
| References | [5], [6] | [7], [8] | [5], [9] |

To include interfacial recombination in the electrical simulation, surface recombination velocities (SRV) were assigned at key material interfaces. In cases where experimental SRV data were unavailable, estimates were made based on similar heterojunctions documented in the literature. These values were kept fixed during simulation and weren't varied parametrically. A full list of

the interface SRVs used in the simulation can be found in Table S3.

**Table S3:** Surface recombination velocities used at different interfaces in the tandem structure

| Interface | Hole surface recombination velocity (cm/s) | Electron surface recombination velocity (cm/s) | References |
|---|---|---|---|
| FTO/$TiO_2$ | $1\times10^5$ | $1\times10^5$ | [10] |
| $TiO_2$/$CsSnGeI_3$ | $1\times10^4$ | $1\times10^4$ | [11], [12] |
| $CsSnGeI_3$/$Cu_2O$ | $5\times10^3$ | $5\times10^3$ | [4] |
| a-Si/GaSb | $1\times10^4$ | $1\times10^4$ | [13] |
| GaSb/c-Si | $1\times10^5$ | $1\times10^5$ | [13] |
| c-Si/a-Si | $1\times10^2$ | $1\times10^2$ | [14] |
| a-Si/$SiO_2$ | $1\times10^2$ | $1\times10^2$ | [14] |

## S2. a-Si Thickness Optimization

The optimal thickness for $CsSnGeI_3$ is 160 nm, while for c-Si, it is 2 µm. These thicknesses were applied to the absorber layers, with the other layer thicknesses kept as previously determined. Variations in thickness were then applied to the n-doped and p-doped a-Si layers in the bottom cell of the tandem structure.

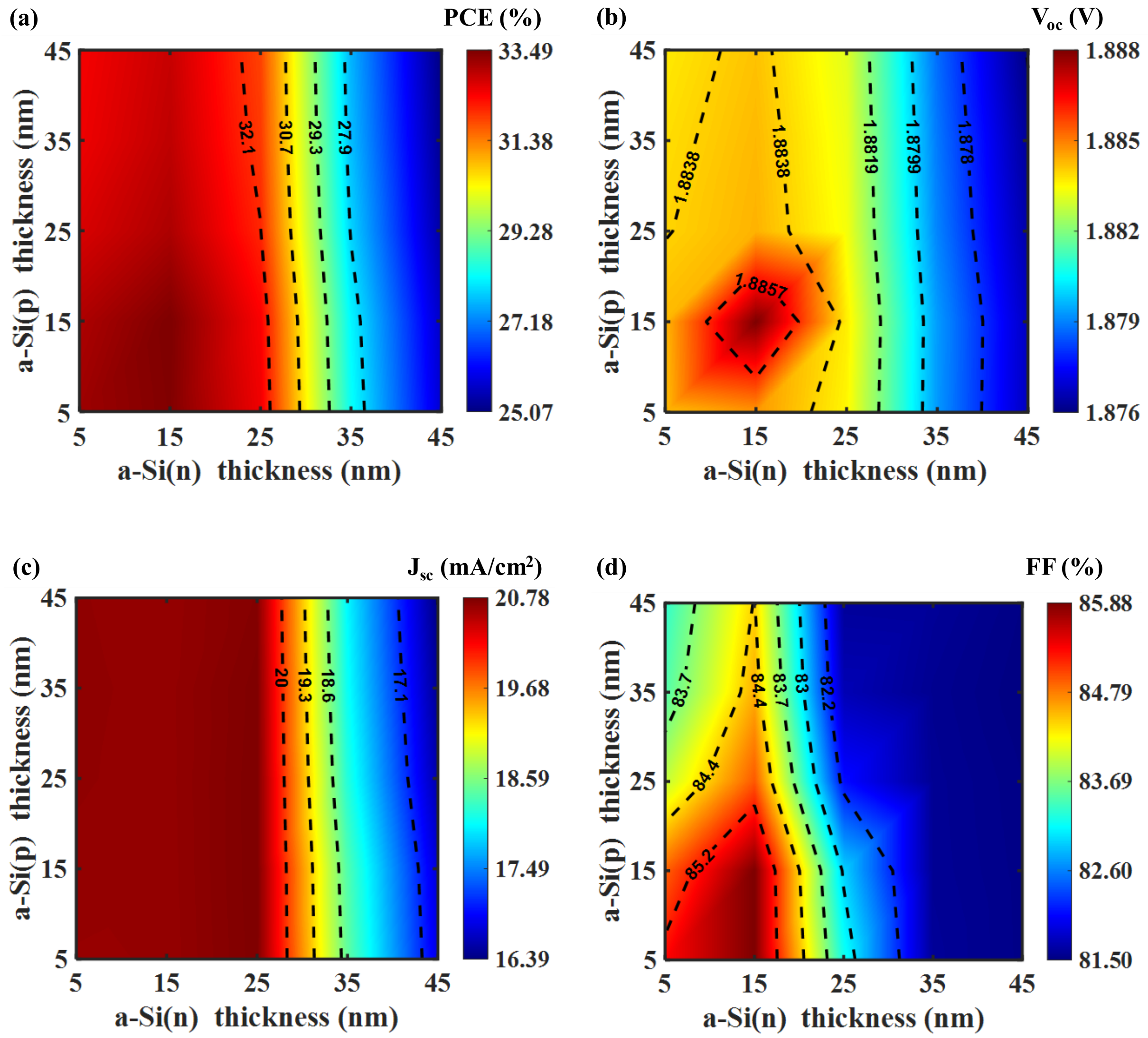


**Figure S1:** Variation of (a) PCE, (b) $V_{oc}$, (c) $J_{sc}$, and (d) FF of tandem cell as a function of thicknesses of a-Si layers of the bottom cell. The highest efficiency is achieved when both the n-doped and p-doped a-Si layer thicknesses are around 15 nm. Notably, PCE and $J_{sc}$ are more responsive to changes in the n-doped a-Si thickness, while the p-doped a-Si thickness has a lesser impact on all metrics.

The plot in fig. S1(a) illustrates that the maximum PCE is reached at a thickness of 15 nm for both types of a-Si layers. As indicated in fig. S1(a), PCE significantly changes with different thicknesses of the n-doped a-Si layer, while it shows only slight variation with changes to the p-doped a-Si layer. Fig. S1(b) shows that the highest $V_{oc}$ occurs at 15 nm for both n-doped and p-doped a-Si layers, confirming the optimal $V_{oc}$ at this thickness. This observation explains the peak

in PCE at 15 nm in fig. S1(a). In fig. S1(c), the $J_{sc}$ of the tandem cell is largely constant concerning variations in the p-doped a-Si layer thickness but displays a clear correlation with the n-doped a-Si layer thickness, with the peak $J_{sc}$ occurring at 15 nm, which supports the optimal PCE. Lastly, fig. S1(d) shows that the FF similarly depends on both a-Si layers, with its highest value at a thickness of 15 nm for each layer. Overall, these results indicate that a thickness of 15 nm is optimal for both a-Si layers, maximizing the tandem cell's overall performance.

## S3. GaSb Thickness Parametric Sweep

After optimizing the perovskite, c-Si, a-Si, ETL, and HTL layers, a thickness sweep was done for the GaSb layer. A thin layer of GaSb was inserted into the bottom cell, creating a SHJ structure. This adjustment enhances absorption in the bottom cell, as GaSb (bandgap 0.72 eV) facilitates the absorption of the solar light spectrum, specially in the NIR region. Consequently, it aids in decreasing the thickness of the c-Si layer in the bottom cell.

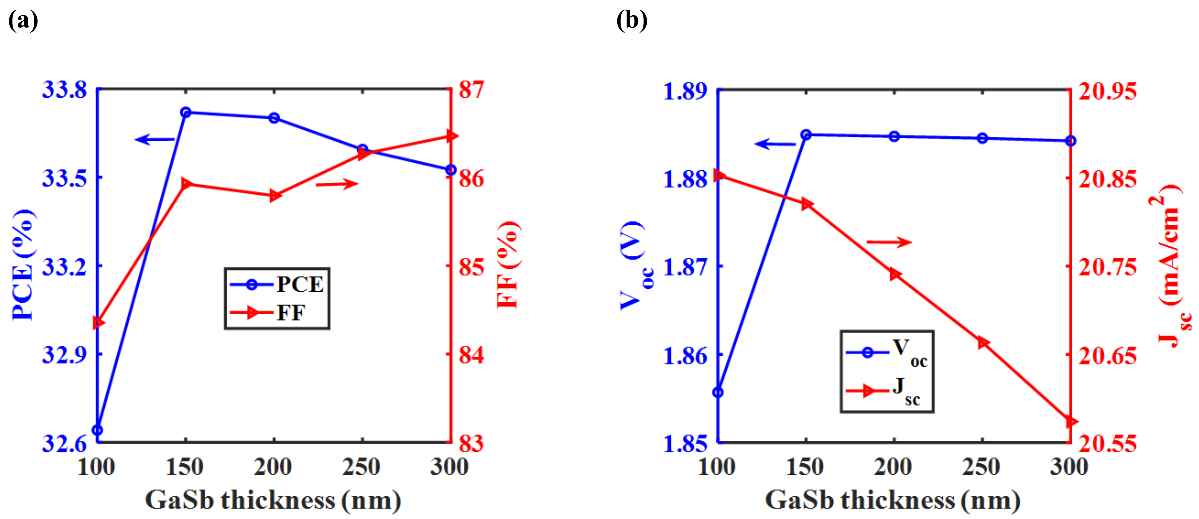


**Figure S2:** Variation of (a) PCE and FF, (b) $V_{oc}$ and $J_{sc}$ of tandem cell as a function of GaSb layer thickness. The optimal device performance occurs at a GaSb thickness of 150 nm, where the PCE and $V_{oc}$ peak, while $J_{sc}$ shows a decreasing trend with increasing GaSb thickness.

Fig. S2 illustrates the effect of the GaSb absorber layer thickness on the performance

metrics of the tandem solar cell. In fig. S2(a), PCE first increases with GaSb thickness, reaching its peak at a thickness of 150 nm. However, the PCE starts to decline beyond this thickness. A similar pattern is evident in fig. S2(b), where the $V_{oc}$ rises up to 150 nm, followed by a slight decrease. Conversely, fig. S2(b) also reveals that the $J_{sc}$ consistently declines as GaSb thickness increases, which points to lower carrier collection efficiency in thicker layers. The FF depicted in fig. S2(a) does not exhibit a straightforward trend. It rises up to 150 nm, decreases at 200 nm, and then increases again as the thickness approaches 300 nm. Overall, the trends observed across all four plots suggest that a GaSb layer thickness of 150 nm achieves the best trade-off between voltage and current behaviour, rendering it the optimal thickness for high-efficiency performance in this tandem setup. Incorporating an ultra-thin GaSb layer at the bottom of the SHJ cell significantly enhanced optical absorption, thereby reducing the required thickness of the c-Si layer.

## S4. ARC Thickness Parametric Sweep

In this study, the ARC layer thickness was also varied to find the ideal thickness for this layer in the tandem structure. $SiO_2$ is used as the ARC layer in this structure.

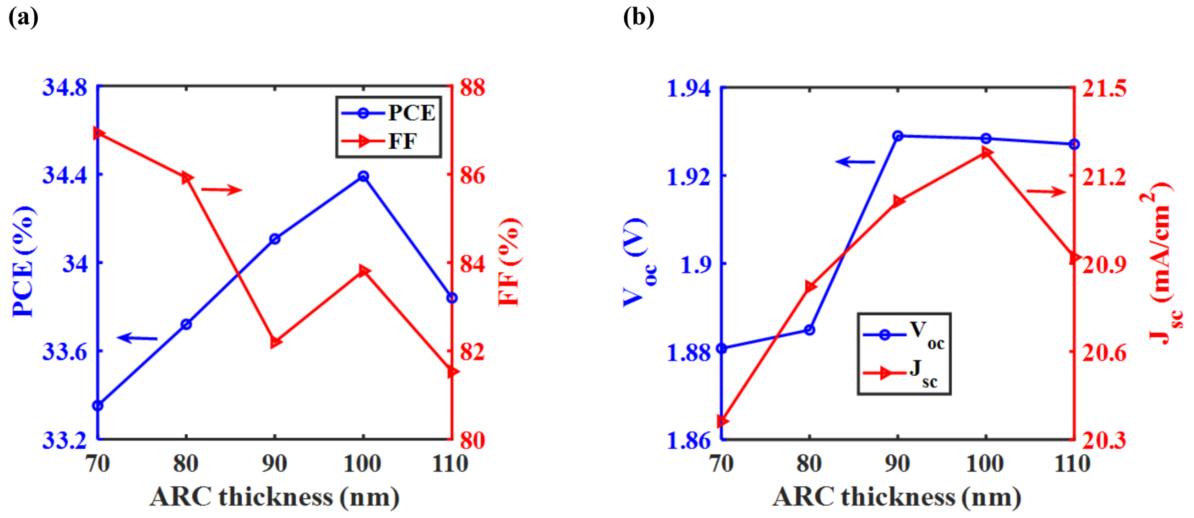


**Figure S3:** Variation of (a) PCE and FF, (b) $V_{oc}$ and $J_{sc}$ of tandem cell as a function of thickness of ARC layer. The ideal thickness of the ARC layer for achieving peak performance metrics is 100 nm. Both PCE and $J_{sc}$ rise with increasing ARC thickness until reaching 100 nm, at which point they reach the highest value. However, $V_{oc}$ experiences a slight decrease when the thickness increases to 100 nm from 90 nm.

Fig. S3(a) illustrates the variation in PCE of the tandem cell with changes in the ARC layer thickness. The PCE increases with ARC thickness up to 100 nm, after which a decline begins. A comparable trend is observed in fig. S3(b), where $J_{sc}$ rises with ARC thickness until it reaches 100 nm, at which point $J_{sc}$ also starts to decrease. However, the ideal value for $V_{oc}$ is at 90 nm thickness as observed in fig. S3(b). But the decrease of $V_{oc}$ from 90 nm to 100 nm is very minimal, not affecting the overall efficiency of the structure. Actually, $V_{oc}$ almost shows a constant value from 90 nm, indicating the dominance of $J_{sc}$ in determining the optimized thickness. Although the FF, shown in fig. S3(a), peaks at 70 nm, the tandem cell's overall performance is best maximized at an ARC thickness of 100 nm. This ARC layer thickness minimizes solar light reflection from the tandem structure, thereby enhancing light absorption and overall device performance.

## S5. Rear Passivation Thickness Parametric Sweep

In the optical simulation, parametric sweeping was used to find the ideal thickness for the

rear passivation layer. The photocurrent densities from both the top and bottom cells were assessed by varying the thickness of the $SiO_2$ rear passivation layer.

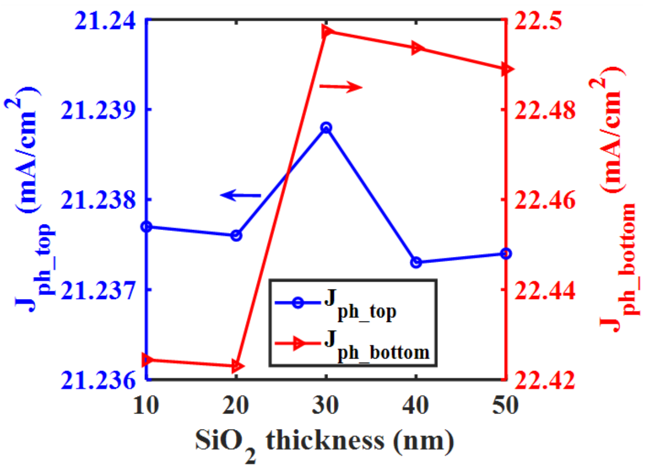


**Figure S4:** Variation of photocurrent density of top cell ($J_{ph\_top}$) and bottom cell ($J_{ph\_bottom}$) as a function of thickness of rear passivation layer. The photocurrent density of the top cell shows minimal variation, with a slight increase at 30 nm, while the bottom cell exhibits a more significant rise at 30 nm, reaching its maximum value at this thickness.

Fig. S4 illustrates how the photocurrent density varies for the top and bottom cells, respectively, depending on the thickness of the rear passivation $SiO_2$ layer. In this figure, the photocurrent density of the top cell peaks at 30 nm thickness, showing a minor fluctuation. However, a more significant increase in photocurrent density is observed for the bottom cell at the same thickness. Thus, 30 nm is the optimal thickness for the $SiO_2$ rear passivation layer.

## S6. Determining the Appropriate Radius of Plasmonic Nanorods

To determine the optimal nanorod radius, photocurrent densities for both the top and bottom cells were evaluated across a range of nanorod sizes using optical simulations.

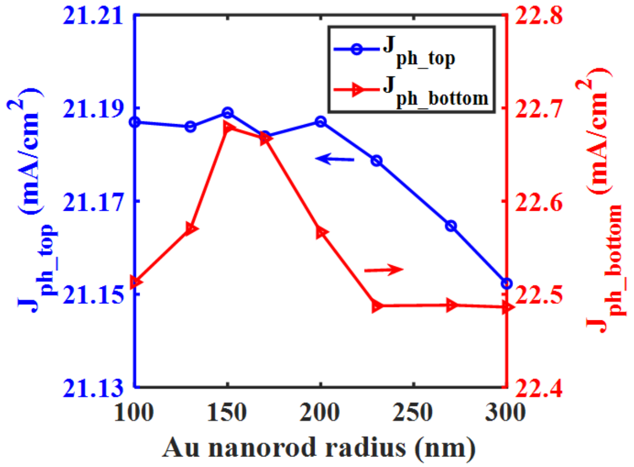


**Figure S5:** Variation of photocurrent density of top cell ($J_{ph\_top}$) and bottom cell ($J_{ph\_bottom}$) as a function of the radius of cylindrical nanorod. An optimal radius of 150 nm is observed, at which the photocurrent densities of both the top and bottom cells peak.

Fig. S5 illustrates how photocurrent density in both the top and bottom cells varies with cylindrical nanorod radius. A peak is observed at 150 nm in both subcells, although the response of the top cell suggests minimal dependence on nanorod geometry. The bottom cell, in contrast, shows a clear increase in photocurrent as the radius approaches 150 nm. This indicates a stronger sensitivity to nanostructure dimensions, likely due to enhanced plasmonic interactions that improve light absorption and charge carrier generation. Overall, this trend confirms that nanorod geometry, particularly radius, can significantly influence optical behavior and should be carefully optimized for maximum efficiency.

## S7. Band Diagram under MPPT Conditions

Band diagrams of the top and bottom cells under MPPT conditions help visualize carrier transport across the tandem structure.

(a)

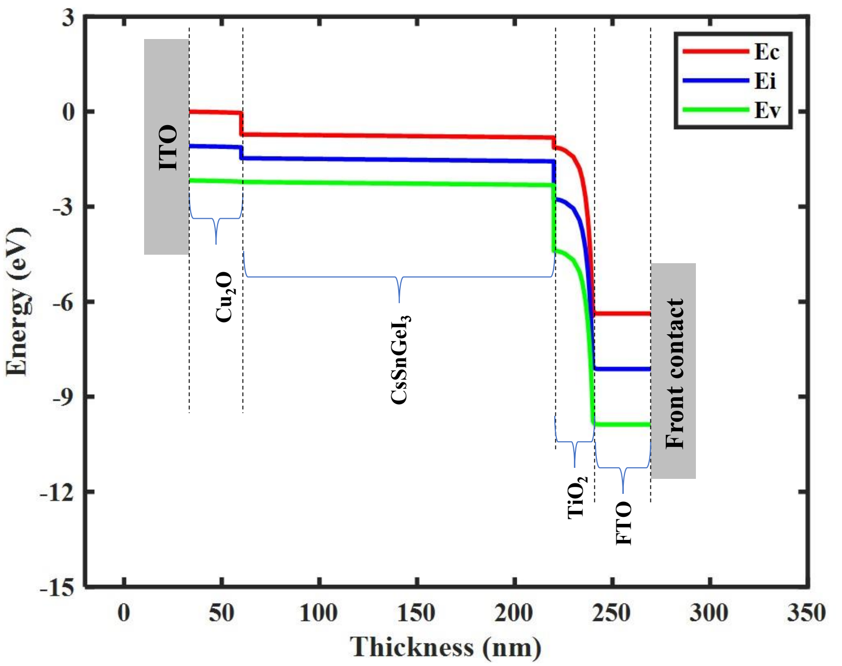


(b)

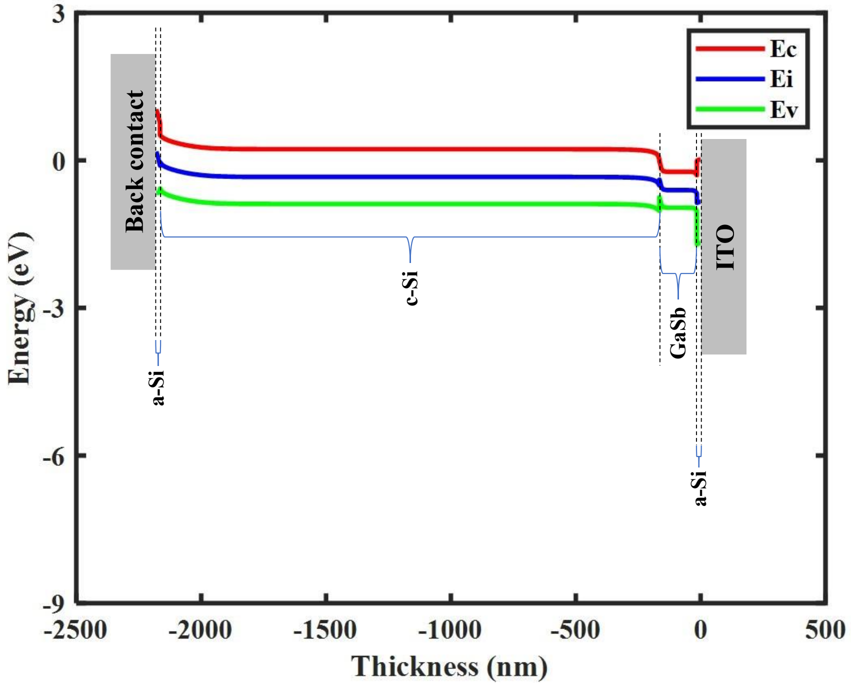


**Figure S6:** Energy band diagram of the (a) top cell and (b) bottom cell under MPPT conditions. The diagrams show the conduction band edge (Ec), intrinsic Fermi level (Ei), and valence band

edge (Ev) across the entire tandem structure, highlighting the seamless transport of charge carriers.

Fig. S6 depicts the energy band diagram of the tandem structure operating under MPPT condition. Ag serves as the back contact, while Al functions as the front contact of the tandem structure. As shown in fig. S6, the seamless transport of both carriers through their respective paths, facilitated by the ITO layer functioning as a recombination layer between the top and bottom cells, contributes to the high efficiency of the tandem structure.

# References


[1] Raghvendra, R. R. Kumar, and S. K. Pandey, "Performance evaluation and material parameter perspective of eco-friendly highly efficient CsSnGeI3 perovskite solar cell," *Superlattices Microstruct*, 2019, vol. 135, 106273, doi: 10.1016/j.spmi.2019.106273.

[2] H. Sabbah, "Numerical Simulation of 30% Efficient Lead-Free Perovskite CsSnGeI3-Based Solar Cells," *Materials*, 2022, vol. 15, no. 9, 3229, doi: 10.3390/ma15093229.

[3] M. T. Islam *et al.*, "Investigation of CsSn0.5Ge0.5I3-on-Si Tandem Solar Device Utilizing SCAPS Simulation," *IEEE Trans Electron Devices*, vol. 68, no. 2, pp. 618–625, Feb. 2021, doi: 10.1109/TED.2020.3045383.

[4] M. K. Hossain *et al.*, "An extensive study on multiple ETL and HTL layers to design and simulation of high-performance lead-free CsSnCl3-based perovskite solar cells," *Sci Rep*, 2023, vol. 13, no. 1, 2521, doi: 10.1038/s41598-023-28506-2.

[5] S. Jamil, U. Saha, and M. K. Alam, "Surface plasmon enhanced ultrathin Cu 2 ZnSnS 4 /crystalline-Si tandem solar cells," *Nanoscale Adv*, vol. 5, no. 11, pp. 2887–2896, May 2023, doi: 10.1039/D2NA00826B.

[6] F. X. A. Abega, A. T. Ngoupo, and J. M. B. Ndjaka, "Numerical Design of Ultrathin Hydrogenated Amorphous Silicon-Based Solar Cell," *International Journal of Photoenergy*, 2021, vol. 2021, 7506837, doi: 10.1155/2021/7506837.

[7] J. Tournet *et al.*, "GaSb-based solar cells for multi-junction integration on Si substrates," *Solar Energy Materials and Solar Cells*, vol. 191, pp. 444–450, Mar. 2019, doi: 10.1016/j.solmat.2018.11.035.

[8] B. Maji and R. Chattopadhyay, "Design and optimization of high efficient GaSb homo-junction solar cell using GaSb intrinsic layer," *Microsystem Technologies*, vol. 27, no. 9, pp. 3589–3598, Sep. 2021, doi: 10.1007/s00542-020-05125-9.

[9] T. Shawky, M. H. Aly, and M. Fedawy, "Performance Analysis and Simulation of c-Si/SiGe Based Solar Cell," *IEEE Access*, vol. 9, pp. 75283–75292, 2021, doi: 10.1109/ACCESS.2021.3080391.

[10] M. A. Green, K. Emery, Y. Hishikawa, W. Warta, and E. D. Dunlop, "Solar cell efficiency tables (Version 45)," *Progress in Photovoltaics: Research and Applications*, vol. 23, no. 1, pp. 1–9, Jan. 2015, doi: 10.1002/PIP.2573.

[11] H. Sabbah, "Numerical Simulation of 30% Efficient Lead-Free Perovskite CsSnGeI3-Based Solar Cells," *Materials 2022, Vol. 15, Page 3229*, vol. 15, no. 9, p. 3229, Apr. 2022, doi: 10.3390/MA15093229.

[12] M. T. Islam *et al.*, "Investigation of CsSn0.5Ge0.5I3-on-Si Tandem Solar Device Utilizing

SCAPS Simulation," *IEEE Trans Electron Devices*, vol. 68, no. 2, pp. 618–625, Feb. 2021, doi: 10.1109/TED.2020.3045383.

[13] J. Tournet *et al.*, "GaSb-based solar cells for multi-junction integration on Si substrates," *Solar Energy Materials and Solar Cells*, vol. 191, pp. 444–450, Mar. 2019, doi: 10.1016/J.SOLMAT.2018.11.035.

[14] S. Sadhukhan *et al.*, "Evaluation of dominant loss mechanisms of PERC cells for optimization of rear passivating stacks," *Surfaces and Interfaces*, vol. 27, p. 101496, Dec. 2021, doi: 10.1016/J.SURFIN.2021.101496.
